# Protective Efficacy of an Inactivated Recombinant Serotype 4 Fowl Adenovirus Against Duck Adenovirus 3 in Muscovy Duck

**DOI:** 10.3390/vaccines12121357

**Published:** 2024-11-30

**Authors:** Yun Lin, Wenjie Jiang, Lihua Ma, Jiayu Zhang, Ye Tang, Qiuqi Kan, Haifeng Xiong, Jiayan Wu, Jun Zhang, Yiwen Niu, Wei Zhang, Shengnan Wang, Weikang Wang, Quan Xie, Tuofan Li, Zhimin Wan, Wei Gao, Hongxia Shao, Aijian Qin, Jianqiang Ye

**Affiliations:** 1 Key Laboratory of Jiangsu Preventive Veterinary Medicine, Key Laboratory for Avian Preventive Medicine, Ministry of Education, College of Veterinary Medicine, Yangzhou University, Yangzhou 225009, China; lyun1994@126.com (Y.L.); 15052506059@163.com (W.J.); ma18994096993@163.com (L.M.); 15852882703@163.com (J.Z.); basttangye@163.com (Y.T.); mx120180738@yzu.edu.cn (Q.K.); dx120220190@stu.yzu.edu.cn (H.X.); dx120230186@stu.yzu.edu.cn (J.W.); 18705279209@163.com (J.Z.); 19513322880@163.com (Y.N.); wangshengnanwsnj@163.com (S.W.); wwkgyy@sina.com (W.W.); 007959@yzu.edu.cn (Q.X.); 007619@yzu.edu.cn (T.L.); wanzm@yzu.edu.cn (Z.W.); wgao@yzu.edu.cn (W.G.); hxshao@yzu.edu.cn (H.S.); aijian@yzu.edu.cn (A.Q.); 2 Jiangsu Co-Innovation Center for Prevention and Control of Important Animal Infectious Diseases and Zoonoses, Yangzhou 225009, China; 3 Joint International Research Laboratory of Agriculture and Agri-Product Safety, The Ministry of Education of China, Yangzhou University, Yangzhou 225009, China; 4 Institutes of Agricultural Science and Technology Development, Yangzhou University, Yangzhou 225009, China; 5 Sinopharm Yangzhou VAC Biological Engineering Co., Ltd., Yangzhou 225127, China; zhangwei21@sinopharm.com

**Keywords:** DAdV-3, FAdV-4, duck, bivalent vaccine, antibody, protective efficacy

## Abstract

Background: Duck adenovirus 3 (DAdV-3) is an emerging pathogen that has caused severe economic losses to the duck industry in China. Recently, the infection of ducks with serotype 4 fowl adenovirus (FAdV-4) has also been reported in China. Therefore, an efficient bivalent vaccine to control the diseases caused by DAdV-3 and FAdV-4 is extremely urgent. In our previous study, a recombinant FAdV-4 expressing Fiber-2 of DAdV-3 was generated and designated as rFAdV-4-Fiber-2/DAdV-3. Methods: Here, the recombinant virus rFAdV-4-Fiber-2/DAdV-3 was inactivated to serve as a bivalent vaccine, and its immunogenicity and protective efficacy against DAdV-3 were evaluated in Muscovy ducks. Results: The subcutaneous injection of rFAdV-4-Fiber-2/DAdV-3 could efficiently induce antibodies against Fiber-2 of DAdV-3 and neutralize antibodies against FAdV-4. After challenges with DAdV-3, in comparison with the non-immunized ducks, the immunized ducks did not show any bodyweight loss, gross lesions, or histopathologic change. Moreover, viral loads in livers and kidneys from immunized ducks were undetectable, whereas those in non-immunized ducks with challenge were significantly high. Conclusions: All these data demonstrate that the inactivated recombinant virus rFAdV-4-Fiber-2/DAdV-3 has the potential to be an efficient vaccine candidate against both FAdV-4 and DAdV-3, although efficacy for FAdV-4 needs to be confirmed experimentally.

## 1. Introduction

Adenoviruses belong to the family *Adenoviridae*, with non-enveloped, double-stranded, and non-segmented DNA genomes [1]. The genomes range from 26 to 45 kb in length and encode three major structural proteins, including Hexon, Penton base, and Fiber [2,3]. The *Adenoviridae* family is currently classified into five genera: *Mastadenovirus*, *Aviadenovirus*, *Atadenovirus*, *Siadenovirus*, and *Ichtadenovirus* [4]. Belonging to *Aviadenovirus*, fowl adenoviruses are divided into five species (A to E) and 12 serotypes (1 to 8a and 8b to 11) [5]. Fowl adenovirus infection can cause inclusion body hepatitis (IBH), hydropericardium hepatitis syndrome (HHS), and gizzard erosion (GE) in poultry [6]. Serotype 4 fowl adenovirus (FAdV-4) is the etiological agent of HHS [7,8,9]. The disease of HHS, characterized by necrosis of the swollen and friable liver and clear or colored liquid in the pericardial sac, was first reported in 1987 in chickens at Angara Goth, Pakistan, and then prevailed in additional countries in Asia, Central America, and South America [6].

FAdV-4 could not only cause diseases in chickens but also infect ducks with varying outcomes in experimental studies [10,11,12,13,14]. In 2015, Chen et al. reported that FAdV-4 caused serious hepatitis and pericardial effusion syndrome in commercial Cherry Valley ducks in Shandong province [15]. In the same year, FAdV-4 infection was also reported from the northeast of China [14]. The affected 45-day-old ducks showed enlarged and discolored livers with severe accumulation of fluid in the pericardium. The disease caused high morbidity and mortality in a commercial duck farm where 200,000 ducks were raised, bringing about serious economic losses.

DAdV-3 was a causal agent of a disease characterized by swollen and hemorrhagic livers and kidneys, resulting in a mortality rate of up to 40% in Muscovy duck flocks [16,17]. Since the outbreak of DAdV-3 infection in 2014, the disease has been widely prevalent in the Jiangsu, Hubei, Anhui, Zhejiang, Jiangxi, Fujian, Guangxi, and Guangdong provinces of China [16,18,19]. Lately, some DAdV-3 stains with truncated ORF67 were reported and spread, becoming the dominant strains in the epidemic areas [16,18]. Moreover, the coinfections of DAdV-3 with other viruses, including duck hepatitis B virus, adeno-associated virus, Muscovy duck-origin goose parvovirus, and Muscovy duck parvovirus, were prevailing, which raised the difficulty of disease prevention and control [18].

To prevent HHS caused by FAdV-4 and increase the economic benefit of the poultry industry, several inactivated, subunit, or attenuated vaccines against FAdV-4 were developed, and their safety and efficacy reflected high clinical application value for disease prevention [8,20]. The vaccine research on the prevention of DAdV-3 infection mainly focused on inactivated and subunit vaccines, and a recent study showed that the Fiber-2 subunit vaccine could induce a strong humoral immune response in Muscovy ducks and provide complete protection against DAdV-3 challenge [21]. Therefore, the vaccine for the prevention of FAdV-4 or DAdV-3 infection has been generated. However, a bivalent vaccine against both FAdV-4 and DAdV-3 is of great value and not available. In our former study, the *fiber-2* gene in FAdV-4 was replaced with the *fiber-2* gene of DAdV-3 to generate a recombinant FAdV-4 (rFAdV-4-Fiber-2/DAdV-3) expressing the *fiber-2* gene of DAdV-3 [22]. The recombinant virus rFAdV-4-DAdV-3-Fiber-2 was efficiently replicated in vitro and highly expressed the Fiber-2 of DAdV-3. In this study, the immunogenicity of rFAdV-4-Fiber-2/DAdV-3 as an inactivated vaccine was assessed, and its protective efficacy against DAdV-3 in Muscovy ducks was evaluated.

## 2. Materials and Methods

### 2.1. Animal Ethics Statement

The Muscovy ducks used in the study were purchased from a commercial hatchery (Yangzhou Guangling Jiumuyuan Animal Husbandry Co., Ltd., Yangzhou, China) in the Jiangsu province of China. The sera from the 1-day-old Muscovy ducks were investigated by blocking ELISA and were recorded to be negative for both DAdV-3 and FAdV-4. All animal experiments were in accordance with institutional animal care guidelines and protocol (SYXY-32), which was approved by the Animal Care Committee of Yangzhou University.

### 2.2. Viruses, Cells, and Antibodies

The viruses DAdV-3, FAdV-4, and rFAdV-4-Fiber-2/DAdV-3 were stored in our laboratory. LMH cells were purchased from the American Type Culture Collection and cultured in Dulbecco Modified Eagle Medium/F12 (Gibco, Grand Island, NY, USA) supplemented with 10% fetal bovine serum (Lonsera, Shanghai, China) at 37 °C with 5% CO_2_. Monoclonal antibody (mAb) 3D9 against Fiber-2 of DAdV-3, mAb 3B5 against Fiber-1 of FAdV-4, and mAb 3D9 labeled with horseradish peroxidase (HRP) were prepared and stored in our laboratory.

### 2.3. Preparation of the Inactivated Recombinant Vaccine

The recombinant virus rFAdV-4-Fiber-2/DAdV-3 (2 × 10^8^ TCID_50_/mL) was inactivated by formaldehyde at 37 °C for 24 h with a final concentration of 0.3%. Afterwards, the inactivated recombinant virus and Tween 80 were mixed with a volume ratio of 96:4 to serve as a water phase, and the white oil and Span 80 were mixed with a volume ratio of 94:6 to serve as an oil phase. Then, the oil phase was added to the water phase with a volume ratio of 3:1, and the liquid mixture was emulsified based on a blender. At the same time, cell culture medium was processed with the same technological process and then served as a negative control vaccine.

### 2.4. Animal Experiment

A total of 36 1-day-old Muscovy ducks were equally and randomly divided into three groups, including a non-immunized and non-challenged group (group NN), a non-immunized and challenged group (group NC), and an immunized and challenged group (group IC). The 3-day-old ducks in the group IC were subcutaneously immunized with the inactivated vaccine (1 × 10^7^ TCID_50_ per duck) through subcutaneous injection in the neck. Meanwhile, ducks in the other two groups were injected with an equal quantity of negative control vaccine. At 7 days post-vaccination (dpv), 14 dpv, and 21 dpv, blood samples were collected and sera were processed for the detection of antibodies against DAdV-3 Fiber-2 using a blocking ELISA. Moreover, a virus neutralization test (VNT) was used for the detection of neutralizing antibodies against both DAdV-3 and FAdV-4. At 14 dpv and 21 dpv, three ducks from each group were randomly selected for weighing. Subsequently, ducks in group NC and group IC were challenged with DAdV-3 (2 × 10^6^ TCID_50_ per duck) through the intramuscular route. Ducks in group NN were injected with an equal quantity of PBS. At 3 days post-challenge (dpc) and 6 dpc, three ducks from each group were euthanized for gross lesion observation, histopathological examination, and viral load determination. At 7 dpc and 14 dpc, three ducks in each group were randomly selected for weighing.

### 2.5. Blocking ELISA

The DAdV-3 Fiber-2 protein (1.25 μg/mL) coating antigen was coated onto a 96-well ELISA plate (100 μL/well) and incubated overnight. The next morning, 5% skim milk was used for the blocking of the plate at 37 °C for 2 h. After the blocking, the quadruple-diluted sera were added into the plate (100 μL/well) for 1 h. Then, the mAb 3D9-HRP (0.59 μg/mL), diluted with a ratio of 1:16000, was incubated with the plate (100 μL/well). The enzyme–substrate reaction was performed with the supplement of TMB single-component substrate solution (Solarbio, Beijing, China) (100 μL/well) and reacted at 37 °C for 10 min. The reaction was terminated by the addition of 2 M H_2_SO_4_ (50 μL/well). The optical density at 450 nm (OD_450nm_) was measured, and the percent inhibition (PI) was calculated by the formula PI (%) = [(OD_450nm_ value of negative serum − OD_450nm_ value of tested serum)/OD_450nm_ value of negative serum] × 100%. Tested serum with a PI value ≥ 18% was determined as positive; otherwise, it was considered to be negative.

### 2.6. Virus Neutralization Test

The collected sera were serially double diluted and then mixed with 100 TCID_50_ of DAdV-3 or FAdV-4 in 200 μL of Dulbecco Modified Eagle Medium/F12 with 1% fetal bovine serum. After the incubation of serum with virus at 37 °C for 1 h, the mixture was incubated with LMH cells in a 96-well plate at 37 °C for 2 h. Then, the LMH cells were washed once and maintained in Dulbecco Modified Eagle Medium/F12 containing 1% fetal bovine serum. After 96 h of culture, the LMH cells were analyzed by indirect immunofluorescent assay (IFA). Briefly, the LMH cells were fixed with prechilled acetone and ethanol (3:2) for 5 min. Then, the cells were incubated with mAb 3D9 or mAb 3B5 as a primary antibody and FITC-conjugated goat anti-mouse IgG (Sigma-Aldrich, St. Louis, MO, USA) as a secondary antibody. The incubation of antibodies with cells was maintained at 37 °C for 45 min. Finally, an inverted fluorescence microscope was used for the observation of fluorescence. The VN titers were determined based on the reciprocals of the highest serum dilution that caused significant inhibition of the specific fluorescence of virus.

### 2.7. Histopathological Examination

Collected livers from the ducks of the three groups were fixed with 10% neutral buffered formalin for 24 h. The fixed liver blocks were embedded in paraffin wax and then cut into sections. The sections, stained with hematoxylin and eosin, were observed under a microscope.

### 2.8. Tissue Viral Load Determination

Collected livers, spleens, and kidneys of the ducks were stored at −80 °C. After thawing, tissues of equal quality (0.1 g) were homogenized with 900 μL PBS. The mixtures were frozen at −80 °C, thawed at room temperature, and then centrifuged at 4 °C. The virus-containing supernatants, processed with the above freezing and thawing cycle two times, were incubated with penicillin–streptomycin at 37 °C for 2 h and then diluted for TCID_50_ titration with LMH cells. The titers of the virus-containing supernatants were measured by the Reed–Muench method using mAb 3D9 through IFA [22].

### 2.9. Statistical Analysis

The statistical analysis was performed with a Student’s *t*-test based on GraphPad 8.0.2 software, and the data were presented as means ± standard deviation. *p* value < 0.05 is considered statistically significant. *, **, ***, and **** mean *p* < 0.05, *p* < 0.01, *p* < 0.001, and *p* < 0.0001, respectively.

## 3. Results

### 3.1. rFAdV-4-Fiber-2/DAdV-3 Induced Efficient Humoral Immune Response in Ducks

**Figure 1:**
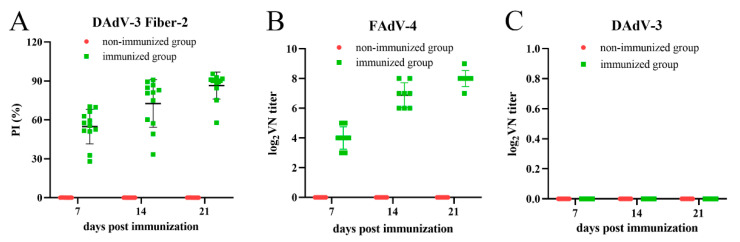
rFAdV-4-Fiber-2/DAdV-3 induced efficient humoral immune response. (**A**): Level of Fiber-2-specific antibodies against DAdV-3 detected by a blocking ELISA. (**B**): Level of neutralizing antibodies against FAdV-4 detected by VNT with LMH cells. (**C**): Level of neutralizing antibodies against DAdV-3 detected by VNT with LMH cells.

Following subcutaneous injection at 3 days of age, antibody response was investigated weekly. As described in Figure 1A, the DAdV-3 Fiber-2-specific antibodies were abundant, with the PI values ranging from 30% to 90%, and the antibody levels at 21 dpv were higher than those at 7 dpv and 14 dpv. To test the neutralizing antibodies against DAdV-3 and FAdV-4, the VNT was performed with the collected sera. As described in Figure 1B, the immunization of the vaccine elicited high-level neutralizing antibodies against FAdV-4, with the titer reaching 2^8^ at 21 dpv. However, the neutralizing antibodies against DAdV-3 were absent, as shown in Figure 1C, although the Fiber-2 specific antibodies were potent. All these data demonstrate that the inactivated rFAdV-4-Fiber-2/DAdV-3 can efficiently induce DAdV-3 Fiber-2-specific antibodies and neutralizing antibodies against FAdV-4, but not against DAdV-3.

### 3.2. rFAdV-4-Fiber-2/DAdV-3 Provided Efficient Protection Against DAdV-3 Challenge

**Figure 2:**
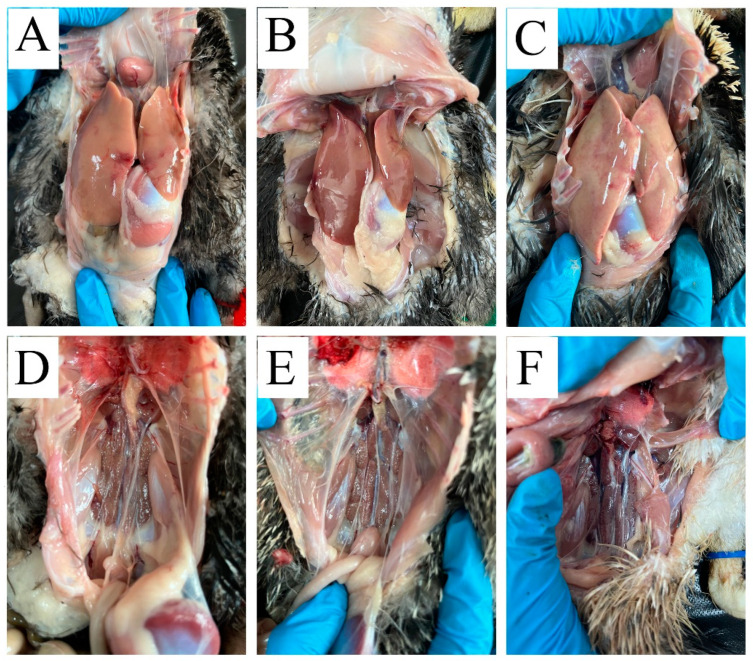
rFAdV-4-Fiber-2/DAdV-3 protected ducks from gross lesions caused by DAdV-3 challenge at 3 dpc. (**A**): Gross lesions of liver from ducks in group NN at 3 dpc. (**B**): Gross lesions of liver from ducks in group IC at 3 dpc. (**C**): Gross lesions of liver from ducks in group NC at 3 dpc. (**D**): Gross lesions of kidney from ducks in group NN at 3 dpc. (**E**): Gross lesions of kidney from ducks in group IC at 3 dpc. (**F**): Gross lesions of kidney from ducks in group NC at 3 dpc.

To evaluate the efficacy of the inactivated vaccine against DAdV-3, ducks in group NC and group IC were intramuscularly challenged with DAdV-3. Then, gross lesions of the ducks in the three groups were investigated at 3 dpc. As shown in Figure 2, ducks in group NN and group IC showed no typical organ damage to livers and kidneys. However, ducks in group NC exhibited significant gross lesions, which were characterized by enlarged yellowish-white livers with hemorrhagic spots and hemorrhaged kidneys. At 6 dpc, ducks in group NC were recovered, and gross lesions of livers and kidneys were as normal as those of ducks in group NN and group IC, as shown in Figure S1.

**Figure 3:**
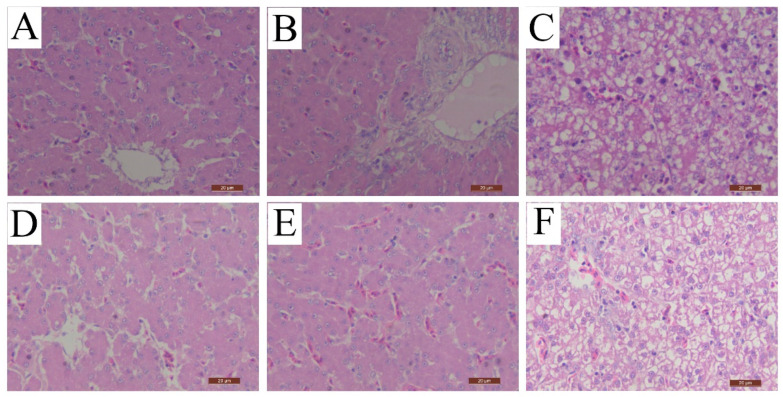
rFAdV-4-Fiber-2/DAdV-3 protected ducks from histopathologic changes caused by DAdV-3 challenge. (**A**): Histopathologic changes of livers from ducks in group NN at 3 dpc. (**B**): Histopathologic changes of livers from ducks in group IC at 3 dpc. (**C**): Histopathologic changes of livers from ducks in group NC at 3 dpc. (**D**): Histopathologic changes of livers from ducks in group NN at 6 dpc. (**E**): Histopathologic changes of livers from ducks in group IC at 6 dpc. (**F**): Histopathologic changes of livers from ducks in group NC at 6 dpc.

To understand the pathological damage of the target organ, histopathological changes of livers were examined. As described in Figure 3C, the hepatocytes of ducks in group NC exhibited steatosis, necrosis, and disintegration at 3 dpc. Furthermore, there were intranuclear inclusion bodies and inflammatory cell infiltrations. At 6 dpc, the typical intranuclear inclusion bodies had disappeared and swollen hepatocytes with inflammation were observed, as shown in Figure 3F. There were no histopathological changes in livers from ducks in group NN and group IC at both time points, as shown in Figure 3A,B,D,E.

**Figure 4:**
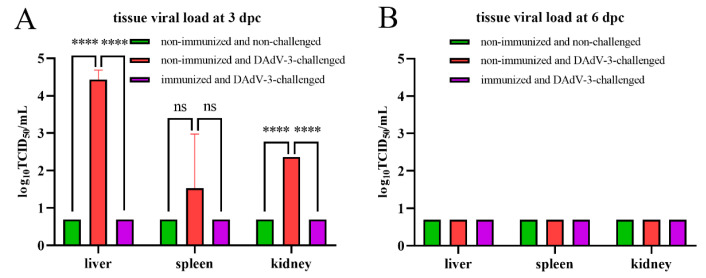
rFAdV-4-Fiber-2/DAdV-3 protected ducks from tissue viral load caused by DAdV-3 challenge. (**A**): Tissue viral loads of livers, spleens, and kidneys from ducks at 3 dpc. (**B**): Tissue viral loads of livers, spleens, and kidneys from ducks at 6 dpc. The experiments were performed with three biological replicates. **** indicates statistically significant with *p* < 0.0001, and ns indicates not statistically significant.

To further evaluate the protection of the inactivated vaccine against DAdV-3, viral titers from livers, spleens, and kidneys were investigated by TCID_50_ in LMH cells. As shown in Figure 4A, no virus was detected in the tissues from ducks in group NN and group IC at 3 dpc, while high titers of virus were revealed from tissues of ducks in group NC. The differences in viral load in livers and kidneys between group NC and the other two groups at 3 dpc were statistically significant. At 6 dpc, no virus was detected from all tissues of ducks in the three groups, as shown in Figure 4B.

**Figure 5:**
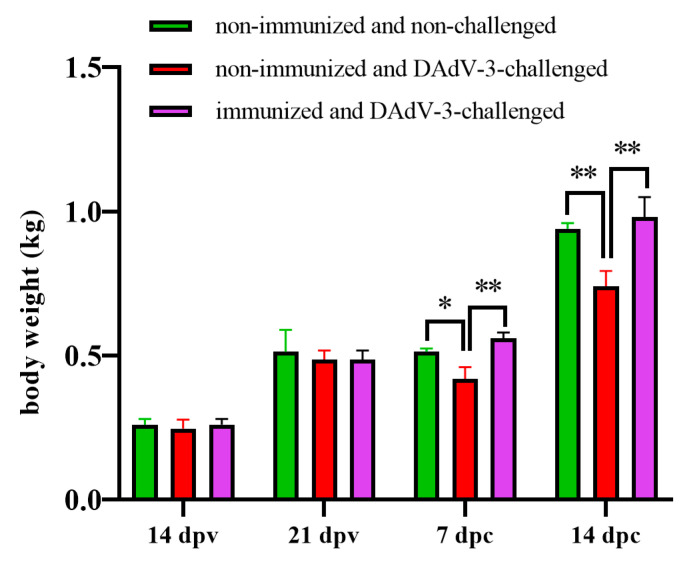
rFAdV-4-Fiber-2/DAdV-3 protected ducks from bodyweight loss caused by DAdV-3 challenge. Three ducks in each group were randomly selected for weighing at 14 dpv, 21 dpv, 7 dpc, and 14 dpc, respectively. * indicates statistically significant with *p* < 0.05, and ** indicates statistically significant with *p* < 0.01.

In addition, the effect of the inactivated vaccine on bodyweight loss was monitored. As shown in Figure 5, the bodyweight of ducks in the three groups had no statistically significant difference at 14 dpv and 21 dpv. Notably, the bodyweight of ducks in group NC was significantly lower than that of ducks in group NN and group IC at both 7 dpc and 14 dpc. All these data highlight that the inactivated rFAdV-4-Fiber-2/DAdV-3 can provide efficient protection against DAdV-3 challenge with high safety.

## 4. Discussion

In recent years, the prevalence of DAdV-3 infection in Muscovy ducks has considerably increased in China, resulting in serious economic losses for the duck farms [19,23]. Notably, FAdV-4 has also been reported to infect ducks naturally [14,15]. Therefore, there is an urgent need to develop a bivalent vaccine for the prevention of the two diseases. Given the fact that Fiber-2 of DAdV-3 is an ideal protective antigen located at the surface of the virion and the feasibility that FAdV-4 is able to efficiently express exogenous protein as a vaccine vector, the recombinant virus rFAdV-4-Fiber-2/DAdV-3 expressing the Fiber-2 of DAdV-3 was generated based on CRISPR/Cas9 technology in our previous work [22]. In this study, the inactivated rFAdV-4-Fiber-2/DAdV-3 was prepared, and its protective efficacy was evaluated in Muscovy ducks through subcutaneous injection.

In this study, the Muscovy ducks were challenged by non-physiological intramuscular injection. It may damage the muscle tissue and blood vessels, which not only causes pain and bleeding, but also increases the infection risk. Moreover, due to the individual difference in the muscle structure and blood circulation status of ducks, intramuscular injection may affect the efficacy of the challenged virus and then weaken the consistency and accuracy of challenging experiments. However, intramuscular injection also possesses significant advantages, such as rapid infection efficacy, simple operation process, and accurate dose control. After the challenge with DAdV-3, the immunized ducks showed full protection with no gross lesions, no histopathological change, and no virus in tissues, which was in agreement with the immune protective effect of Fiber-2 subunit vaccine against DAdV-3 [21]. In comparison, the non-immunized ducks suffered from serious tissue damages resulting from the challenge of DAdV-3 at the early stage. To our surprise, the non-protected ducks recovered at 6 dpc with mild lesions and no virus detectable. Some studies indicated the high pathogenicity of DAdV-3 [17,19,23]. Notably, the ages of Muscovy ducks used in these studies were 5-day-old, 8-day-old, and 10-day-old, respectively. However, in our study, the age of ducks challenged with DAdV-3 was 24-day-old. Therefore, the age of ducks may affect the pathogenicity of the virus used in our study. Although the non-immunized ducks recovered well from the tissue damages, the bodyweight loss caused by DAdV-3 challenge was not compensated until the end of the study. Notably, the immunization of the inactivated vaccine could protect the ducks from the loss of bodyweight completely, contributing to significant economic benefits.

The inactivated vaccine is capable of inducing humoral immunity, which is crucial for virus elimination and disease prevention. Similar to previous studies, the ducks immunized with the inactivated rFAdV-4-Fiber-2/DAdV-3 elicited high-level neutralizing antibodies against FAdV-4, suggesting that it might prevent against an FAdV-4 infection as well, which needs to be demonstrated [24,25]. In terms of DAdV-3, the immunized ducks induced abundant antibodies against Fiber-2 of DAdV-3; however, no neutralizing antibodies against DAdV-3 could be detected, which was inconsistent with previous reports that the Fiber-2 of DAdV-3 expressed in *Escherichia coli* and used as a subunit vaccine induced weak neutralizing antibodies [21]. Anyhow, the study revealed that the immunity of Fiber-2 provided protection against the pathogenic effects of DAdV-3 challenge in vivo. Notably, the observed protective effect did not exclusively rely on virus-neutralizing activity of Fiber-2 antibodies, which was similar to the protective effect of immunity of FAdV-4 Fiber-2 against FAdV-4 [26,27]. Several studies indicated that cellular immune response could be activated not only by live vaccines [28,29], but also by Fiber subunit vaccines [30,31,32,33]. The vaccination of a chimeric Fiber protein induced a strong local cell-mediated immune response, which played a substantial role in protecting chickens from HHS [30]. Moreover, the importance of non-neutralizing antibodies for the protection of vaccines against avian influenza virus has been proven [34,35,36,37]. Therefore, the non-neutralizing antibodies may play an important part in the immune protection of DAdV-3 Fiber-2. Those non-neutralizing antibodies may enhance cellular immune response by means of antibody-dependent cellular cytotoxicity (ADCC) or antibody-dependent cellular phagocytosis (ADCP), which needs to be further verified experimentally.

In addition, the protective efficacy of the inactivated rFAdV-4-Fiber-2/DAdV-3 against FAdV-4 was evaluated abortively in our previous study because FAdV-4 failed to infect Muscovy ducks through the intramuscular injection. The Muscovy ducks injected with FAdV-4 had no gross lesions, no virus in tissues, and no specific antibody. Pan et al. and Li et al. also reported that FAdV-4 infection could not cause clinical signs and gross lesions in ducks experimentally, which was in accord with our results [11,14]. Contrastingly, Tang et al. noticed an impact on bodyweight loss, and Yu et al. reported a typical hydropericardium and hepatitis in ducks following FAdV-4 infection [10,13]. In addition, Wu et al. reported a high mortality in the experimental study on the pathogenicity of FAdV-4 to ducks [12]. Overall, the experimental data on FAdV-4 infection in ducks were controversial, which might result from the genomic differences between FAdV-4 strains isolated from diseased ducks and from chickens.

## 5. Conclusions

In summary, this is a demonstration of an efficient vaccine candidate rFAdV-4-Fiber-2/DAdV-3, which induced efficient humoral antibodies and provided full protection against DAdV-3 in spite of the lack of neutralizing antibodies. Further efforts need to be made to investigate the protective mechanism of rFAdV-4-Fiber-2/DAdV-3 against DAdV-3.

## Data Availability

The datasets used and analyzed in the study are available from the corresponding author on reasonable request.

## Supplementary Materials

The following supporting information can be downloaded at https://www.mdpi.com/article/10.3390/vaccines12121357/s1. Figure S1: Gross lesions of immunized and non-immunized ducks challenged with DAdV-3 at 6 dpc.

## References

[1] Schachner A., Hess M. (2022). Special issue: Avian adenoviruses. Viruses.

[2] Wang K.L., Liu C., Du X.S., Ma Y., Chen L.L., Cao S.L., Lu J.B., Li Y.B., Si Z.S. (2023). Complete genome sequence and pathogenicity analysis of a highly pathogenic FAdV-4 strain. Res. Vet. Sci..

[3] Yin D.D., Xue M., Yang K.K., Xiong X.Q., Geng S., Tu J., Song X.J., Shao Y., Wang G.J., Qi K.Z. (2020). Molecular characterization and pathogenicity of highly pathogenic fowl adenovirus serotype 4 isolated from laying flock with hydropericardium-hepatitis syndrome. Microb. Pathog..

[4] Benko M., Aoki K., Arnberg N., Davison A.J., Echavarria M., Hess M., Jones M.S., Kajan G.L., Kajon A.E., Mittal S.K. (2022). ICTV virus taxonomy profile: Adenoviridae 2022. J. Gen. Virol..

[5] Hess M. (2000). Detection and differentiation of avian adenoviruses: A review. Avian Pathol..

[6] Schachner A., Matos M., Grafl B., Hess M. (2018). Fowl adenovirus-induced diseases and strategies for their control—A review on the current global situation. Avian Pathol..

[7] Asthana M., Chandra R., Kumar R. (2013). Hydropericardium syndrome: Current state and future developments. Arch. Virol..

[8] El-Shall N.A., El-Hamid H.S.A., Elkady M.F., Ellakany H.F., Elbestawy A.R., Gado A.R., Geneedy A.M., Hasan M.E., Jaremko M., Selim S. (2022). Epidemiology, pathology, prevention, and control strategies of inclusion body hepatitis and hepatitis-hydropericardium syndrome in poultry: A comprehensive review. Front. Vet. Sci..

[9] Li P.H., Zheng P.P., Zhang T.F., Wen G.Y., Shao H.B., Luo Q.P. (2017). Fowl adenovirus serotype 4: Epidemiology, pathogenesis, diagnostic detection, and vaccine strategies. Poult. Sci..

[10] Yu X.L., Wang Z.Z., Chen H., Niu X.Y., Dou Y.G., Yang J., Tang Y., Diao Y.X. (2018). Serological and pathogenic analyses of fowl adenovirus serotype 4 (FAdV-4) strain in Muscovy ducks. Front. Microbiol..

[11] Li R., Li G., Lin J., Han S.J., Hou X.L., Weng H.Y., Guo M.J., Lu Z., Li N., Shang Y.L. (2018). Fowl adenovirus serotype 4 SD0828 infections causes high mortality rate and cytokine levels in specific pathogen-free chickens compared to ducks. Front. Immunol..

[12] Wu B., Yang B., He D., Tang Y., Diao Y. (2022). Genetic evolution of fowl adenovirus serotype 4 and its pathogenicity to Cherry Valley ducks in China. Vet. Microbiol..

[13] Tang Z.H., Liu M., Gao Z.S., Li M.C., Cao J., Ye H.L., Song S.Q., Yan L.P. (2022). Pathogenicity and virus shedding ability of fowl adenovirus serotype 4 to ducks. Vet. Microbiol..

[14] Pan Q., Liu L., Wang Y., Zhang Y., Qi X., Liu C., Gao Y., Wang X., Cui H. (2017). The first whole genome sequence and pathogenicity characterization of a fowl adenovirus 4 isolated from ducks associated with inclusion body hepatitis and hydropericardium syndrome. Avian Pathol..

[15] Chen H., Dou Y., Zheng X., Tang Y., Zhang M., Zhang Y., Wang Z., Diao Y. (2017). Hydropericardium hepatitis syndrome emerged in Cherry Valley ducks in China. Transbound. Emerg. Dis..

[16] Chu L., Ye S., Wang J., Peng D., Wang X., Qian Y., Zhang D. (2022). An insertion and deletion mutant of adenovirus in Muscovy ducks. Arch. Virol..

[17] Zhang X., Zhong Y., Zhou Z., Liu Y., Zhang H., Chen F., Chen W., Xie Q. (2016). Molecular characterization, phylogeny analysis and pathogenicity of a Muscovy duck adenovirus strain isolated in China in 2014. Virology.

[18] Yin L., Zhou Q., Mai K., Yan Z., Shen H., Li Q., Chen L., Zhou Q. (2022). Epidemiological investigation of duck adenovirus 3 in southern China, during 2018–2020. Avian Pathol..

[19] Shi S.H., Liu R.C., Wan C.H., Cheng L.F., Chen Z., Fu G.H., Chen H.M., Fu Q.L., Huang Y. (2019). Isolation and characterization of duck adenovirus 3 circulating in China. Arch. Virol..

[20] Liu A.J., Zhang Y., Cui H.Y., Wang X.M., Gao Y.L., Pan Q. (2022). Advances in Vaccine Development of the Emerging Novel Genotype Fowl Adenovirus 4. Front. Immunol..

[21] Yin L., Chen L., Luo Y., Lin L., Li Q., Peng P., Du Y., Xu Z., Xue C., Cao Y. (2019). Recombinant fiber-2 protein protects Muscovy ducks against duck adenovirus 3 (DAdV-3). Virology.

[22] Guo Y., Lin Y., Xie Q., Zhang W., Xu Z., Chao Y., Cao X., Jiang H., Li H., Li T. (2023). A novel recombinant serotype 4 fowl adenovirus expressing fiber-2 protein of duck adenovirus 3. Front. Cell. Infect. Microbiol..

[23] Shi X.J., Zhang X.Y., Sun H.W., Wei C.Q., Liu Y.N., Luo J.G., Wang X.B., Chen Z.Y., Chen H.J. (2022). Isolation and pathogenic characterization of duck adenovirus 3 mutant circulating in China. Poult. Sci..

[24] Lu H., Xie Q., Zhang W., Zhang J.J., Wang W.K., Lian M.J., Zhao Z.H., Ren D., Xie S.H., Lin Y. (2022). A novel recombinant FAdV-4 virus with Fiber of FAdV-8b provides efficient protection against both FAdV-4 and FAdV-8b. Viruses.

[25] Wang B.Y., Song M.Z., Song C.C., Zhao S.Y., Yang P.P., Qiao Q.L., Cong Y.F., Wang Y.L., Wang Z., Zhao J. (2022). An inactivated novel chimeric FAdV-4 containing fiber of FAdV-8b provides full protection against hepatitis-hydropericardium syndrome and inclusion body hepatitis. Vet. Res..

[26] De Luca C., Schachner A., Heidl S., Hess M. (2022). Vaccination with a fowl adenovirus chimeric fiber protein (crecFib-4/11) simultaneously protects chickens against hepatitis-hydropericardium syndrome (HHS) and inclusion body hepatitis (IBH). Vaccine.

[27] Schachner A., Marek A., Jaskulska B., Bilic I., Hess M. (2014). Recombinant FAdV-4 fiber-2 protein protects chickens against hepatitis-hydropericardium syndrome (HHS). Vaccine.

[28] Schonewille E., Singh A., Gobel T.W., Gerner W., Saalmuller A., Hess M. (2008). Fowl adenovirus (FAdV) serotype 4 causes depletion of B and T cells in lymphoid organs in specific pathogen-free chickens following experimental infection. Vet. Immunol. Immunopathol..

[29] Schonewille E., Jaspers R., Paul G., Hess M. (2010). Specific-pathogen-free chickens vaccinated with a live FAdV-4 vaccine are fully protected against a severe challenge even in the absence of neutralizing antibodies. Avian Dis..

[30] De Luca C., Schachner A., Heidl S., Hess M., Liebhart D., Mitra T. (2022). Local cellular immune response plays a key role in protecting chickens against hepatitis-hydropericardium syndrome (HHS) by vaccination with a recombinant fowl adenovirus (FAdV) chimeric fiber protein. Front. Immunol..

[31] De Luca C., Schachner A., Mitra T., Heidl S., Liebhart D., Hess M. (2020). Fowl adenovirus (FAdV) fiber-based vaccine against inclusion body hepatitis (IBH) provides type-specific protection guided by humoral immunity and regulation of B and T cell response. Vet. Res..

[32] Chen L., Yin L., Zhou Q., Li Q., Luo Y., Xu Z., Zhang Y., Xue C., Cao Y. (2018). Immunogenicity and protective efficacy of recombinant fiber-2 protein in protecting SPF chickens against fowl adenovirus 4. Vaccine.

[33] Gupta A., Ahmed K.A., Ayalew L.E., Popowich S., Kurukulasuriya S., Goonewardene K., Gunawardana T., Karunarathna R., Ojkic D., Tikoo S.K. (2017). Immunogenicity and protective efficacy of virus-like particles and recombinant fiber proteins in broiler-breeder vaccination against fowl adenovirus (FAdV)-8b. Vaccine.

[34] Hu Z., Zhao J., Zhao Y., Fan X., Hu J., Shi L., Wang X., Liu X., Hu S., Gu M. (2019). Hemagglutinin-specific non-neutralizing antibody is essential for protection provided by inactivated and viral-vectored H7N9 avian influenza vaccines in chickens. Front. Vet. Sci..

[35] Sicca F., Neppelenbroek S., Huckriede A. (2018). Effector mechanisms of influenza-specific antibodies: Neutralization and beyond. Expert Rev. Vaccines.

[36] Stadlbauer D., Rajabhathor A., Amanat F., Kaplan D., Masud A., Treanor J.J., Izikson R., Cox M.M., Nachbagauer R., Krammer F. (2017). Vaccination with a recombinant H7 hemagglutinin-based influenza virus vaccine induces broadly reactive antibodies in humans. Msphere.

[37] Dunand C.J.H., Leon P.E., Huang M., Choi A., Chromikova V., Ho I.Y., Tan G.S., Cruz J., Hirsh A., Zheng N.Y. (2016). Both neutralizing and non-neutralizing human H7N9 influenza vaccine-induced monoclonal antibodies confer protection. Cell Host Microbe.

