# Supplementary material for: Protective Efficacy of an Inactivated Recombinant Serotype 4 Fowl Adenovirus Against Duck Adenovirus 3 in Muscovy Duck
Source: Vaccines (Basel). 2024 Nov 30;12(12):1357. doi: 10.3390/vaccines12121357 (PMC11680251; doi:10.3390/vaccines12121357)
Supplement: Supplementary file 1 [file vaccines-12-01357-s001.zip › vaccines-3317329-supplementary.pdf]

vaccines

Supplementary Materials

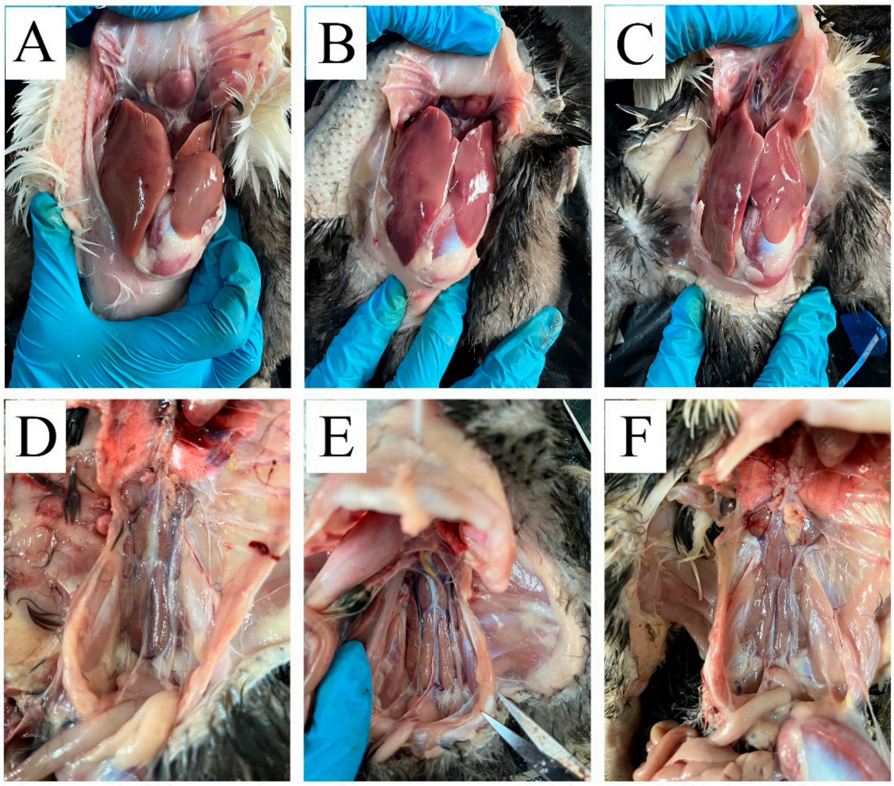

Figure S1. Gross lesions of immunized and non-immunized ducks challenged with DAdV-3 at 6 dpc. A: Gross lesions of liver from duck in group NN at 6 dpc. B: Gross lesions of liver from duck in group IC at 6 dpc. C: Gross lesions of liver from duck in group NC at 6 dpc. D: Gross lesions of kidney from duck in group NN at 6 dpc. E: Gross lesions of kidney from duck in group IC at 6 dpc. F: Gross lesions of kidney from duck in group NC at 6 dpc.
